# Can’t Afford It

**Published:** 1862-06

**Authors:** 


### CAN'T AFFORD IT.

A PROFESSIONAL (!) brother said to us, after we had pointed out the excellencies of a new work upon the science and practice of our profession, that he should be much pleased to have the work, and indeed all such works, and the journals too, but he could not afford it. Upon a little investigation, the reasons he could not afford it were very palpable. Upon inquiry, we found that his library consisted of the following valuable works, viz.: Fitch's Dental Surgery, Bell on the Teeth, Goddard on the Teeth, one of the first editions of Harris' Principles and Practice, and a few scattering numbers of the Dental Recorder, News Letter, The Dental Lamp; and he remarked that he always got the catalogues from the depots. Now, it is plain enough that any one having such a library, and being fully posted in all its science and lore, couldn't afford to buy any more books; it is a wonder how such a man can afford to live. The only way in which he can afford to live is by practicing among a people more ignorant than himself. The means of professional knowledge are at best very limited, even when we gather up and make available all that is extant; and he who has not grown by such means for the last fifteen or twenty years is certainly very much dwarfed, indeed, he has never got out of the shell, and somebody ought to crack it for him, and help him out, if he would be worth anything after his liberation.

This same class of men can't afford to attend dental associations, nor indeed any of the ordinary means of improvement; they can't afford to do anything, except hang on to the profession, like loathsome, dead excrescences, that should have been shaken off long ago. Any man who has the will to avail himself of the professional advantages within his reach can afford to do it, and it will be to his advantage as a man, as a dentist, as a citizen, and as a Christian, and immeasurably to the advantage of his patients.

